# Revealing Rembrandt

**DOI:** 10.3389/fnins.2014.00076

**Published:** 2014-04-21

**Authors:** Andrew J. Parker

**Affiliations:** Department of Physiology, Anatomy and Genetics, University of OxfordOxford, UK

**Keywords:** art, Rembrandt, fMRI, authenticity, cortical response properties

## Abstract

The power and significance of artwork in shaping human cognition is self-evident. The starting point for our empirical investigations is the view that the task of neuroscience is to integrate itself with other forms of knowledge, rather than to seek to supplant them. In our recent work, we examined a particular aspect of the appreciation of artwork using present-day functional magnetic resonance imaging (fMRI). Our results emphasized the continuity between viewing artwork and other human cognitive activities. We also showed that appreciation of a particular aspect of artwork, namely authenticity, depends upon the co-ordinated activity between the brain regions involved in multiple decision making and those responsible for processing visual information. The findings about brain function probably have no specific consequences for understanding how people respond to the art of Rembrandt in comparison with their response to other artworks. However, the use of images of Rembrandt's portraits, his most intimate and personal works, clearly had a significant impact upon our viewers, even though they have been spatially confined to the interior of an MRI scanner at the time of viewing. Neuroscientific studies of humans viewing artwork have the capacity to reveal the diversity of human cognitive responses that may be induced by external advice or context as people view artwork in a variety of frameworks and settings.

Shortly after the Tate Modern art gallery in London opened, it was reported that more people in the UK went to museums and galleries than attended football matches (Travers, 2006). At more than 5 million people in 2012 alone for the exhibits of modern art in the Tate, the level of interest is evident. Of course, crude numbers do not tell us anything directly about the quality of the art on exhibit, but a number like this is impressive to a biologist. With more than 20 million visitors in 5 years, a sizable fraction of the UK population has visited this one location.

Each person who goes to a gallery such as the Tate expends a good deal of resources (money, food, time) simply to view objects. Biologists would always consider behavioral activity of this kind as indicative of fundamental choices. If resources are being expended on visiting galleries and viewing art, they are not available for other competing activities. Considered merely as materials, the art objects in the gallery are not very valuable in themselves. They are predominantly composed of wood, canvas, plastic, acrylic or oil paints, glass, stone, concrete, fabrics, and so on. These are all common-place materials, well within the household budgets of the visitors. Objects like Damien Hirst's diamond-encrusted skull are exceptions, holding some of their shock value as artworks because they depart from normality.

What is special about art objects is the way in which the materials have been configured to make certain visual impressions. These impressions are clearly attractive and meaningful to lots of people: the numbers above are enough to convince of this. Biologists are then obliged to think about this in the following way. The impressions created by the artworks are sufficiently valued as experiences to compensate for the expenditure of resources (time, effort, money), bearing in mind that these resources could be equally well-applied to serving basic biological needs such as nutrition or reproduction. There is a choice to be made and many people are choosing to expend their biological resources on viewing artwork.

There are several factors that combine to bring about this situation. First, the resources expended on viewing artwork are not needed for allocation to basic biological needs. Second, the reward, pleasure or excitement from viewing artwork is sufficient to merit a claim upon a fraction of the available resources, in competition with other possible activities. In mentioning reward or pleasure here, we do not want to be limited to positive affective states. Art also conveys and manipulates negative emotional responses, such as fear, disgust, and anxiety, and works of art that successfully excite viewers in this way gain a great deal of attention and analysis. For both positive and negative affective states, choice is at the center of this framework, such that decisions are made to engage in a limited number of activities out of a potentially wider set that is available.

The other component of people's choices that is of interest to biologists is the visual content itself. Put simply, what is special about the visual content of artworks? Is the special nature of artworks fully captured by the visual impressions that they create? Questions such as this are superficially simple but become more complex as we think about possible answers. Certainly, the visual neuroscientist will want to know whether the visual impressions created by artwork are in anyway distinguishable from the visual impressions created by objects or scenes that are not considered to be artwork. There probably is no hard boundary between objects that are “art” and those that are not, but this does not make the question unapproachable. Figure 1 illustrates one of the uncomfortable issues that might be posed in this domain. Each of the half-images presents a portion of an artistic work that is plausibly a portrait by Rembrandt: indeed the each of the original paintings has at some time been accepted as a genuine Rembrandt. The one on the left is currently acknowledged as genuine, whereas the one on the right is now more disputed. Clearly, the visual impressions from the disputed portrait do not vary as the experts argue back and forth; however, equally clearly, the portrait's status as an artwork is affected by the outcome of these discussions.

**Figure 1:**
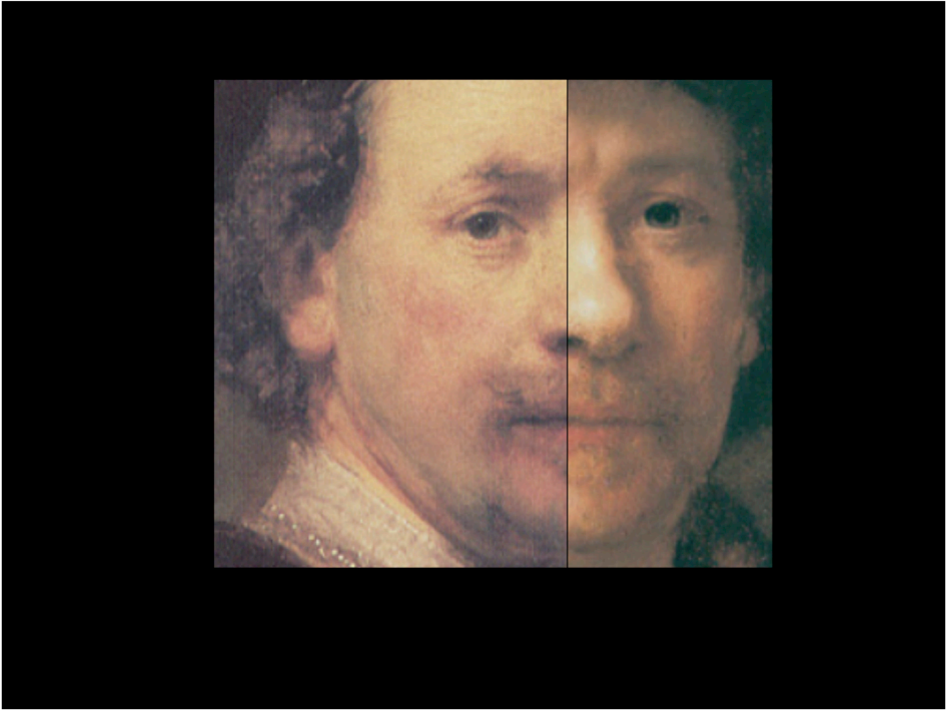
**A chimera image of a portions of a pair of Rembrandt self-portraits: one half image is from a genuine self-portrait by Rembrandt (Norton Simon Museum of Fine Art, Pasadena); the other half image is from a portrait attributed to Rembrandt (Museo Thyssen-Bornemisza, Madrid), whose authenticity has been hotly debated among experts for more than 40 years, see pp. 361–370 in (van de Wetering, 2005)**. Chimera image prepared by Martin Kemp.

Turning the question the other way, there has been a great deal of interest broadly within arts and humanities about whether neuroscientific knowledge, particularly cognitive neuroscience, offers insights into the questions that have been their long-standing preoccupations: literary theory, artistic appreciation, theory of knowledge, philology and linguistics, and so forth. Debating these issues is not simply an abstracted and theoretical discussion, removed from everyday concerns. One may point to the role of psychology and cognitive neuroscience in shaping our understanding of human memory and recollection. This understanding has fed into practical situations such as the reliability of witnesses in legal cases. Personal statements from witnesses, based on their presumed faithful recollections, would have been at the center of criminal prosecutions some 50–60 years ago, whereas now the rise of forensic technology and the demonstrated unreliability of human memory under some circumstances have promoted a shift away from the centrality of personal witness.

## Neuroscience and art

The attempt to fuse neuroscientific insights with mainstream art theory has led in a number of directions, not all of them welcomed by scholars of art history and critical theory. The most direct confrontation has been generated over the idea that neuroscientists might be able to identify a specific region of the brain that is associated with the appreciation of beauty. For example, Kawabata and Zeki (2004) asked people to rate different artworks (paintings) as beautiful, neutral, or ugly; the participants then viewed those same paintings whilst lying inside a magnetic resonance imaging system (brain scanner) that registered the changes in functional blood flow that arise as different parts of the brain's neural tissue becomes active. The authors found that different parts of the brain were activated by images rated as beautiful in comparison with images rated as ugly, although some other brain regions were activated by beautiful or ugly but not by neutral images. This approach can be imaginatively extended to include less familiar esthetic judgments. Recent work has approached the question of why and how mathematically sophisticated observers find certain mathematical expressions to be elegant and esthetically pleasing (Zeki et al., 2014).

This work has been extended away from **functional magnetic resonance imaging (fMRI)** (Munar et al., 2012) with a variety of approaches, some of which have looked at gender differences in the brain responses (Cela-Conde et al., 2009), whereas others have sought to peel apart different aspects of the visual experience, examining the link between visual symmetry and beauty (Jacobsen et al., 2006) and contrasting the judgment of beauty with that of “naturalness” and more direct physical qualities of the visual object, such as surface roughness (Jacobs et al., 2012). Some of these studies have used visual objects that are certainly interesting and engaging to look at but might not qualify as art-works by most standards, in that they are images generated by a computer, rather than created by a person—of course, there can be art produced with computing techniques, but the resulting products (images, sounds, 3-D printed objects) pass through a stage of appraisal by the artist, followed by rejection or selection.

KEY CONCEPT 1. Functional magnetic resonance imaging (fMRI) Functional magnetic resonance imaging (fMRI) is a technique that measures the oxygenation level of cerebral blood flow to make inferences about neural activity changes that are localized to particular sites in the brain.

**Figure 2:**
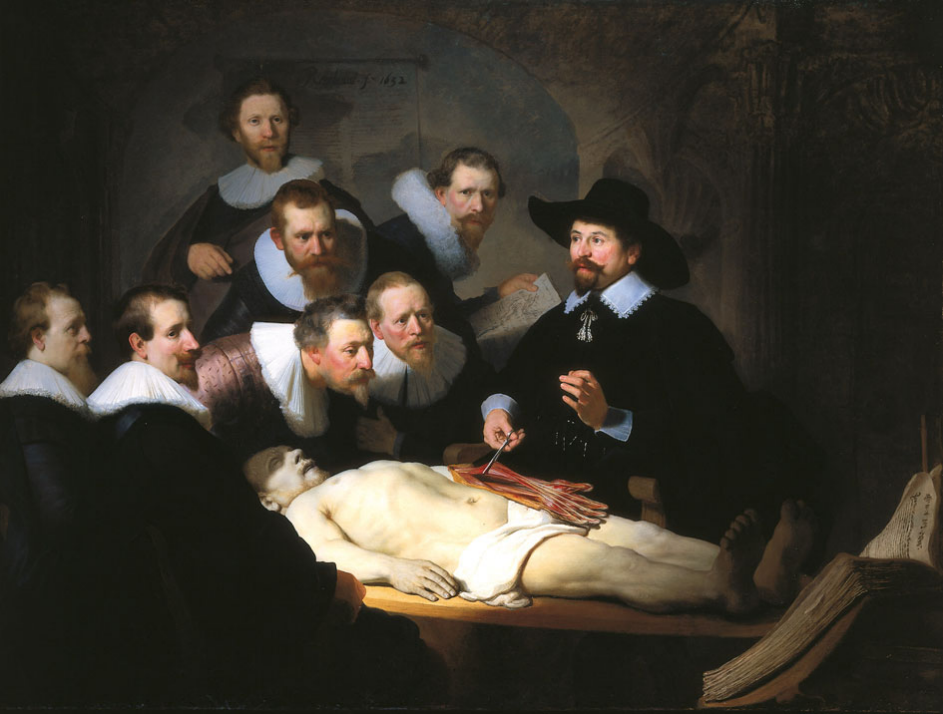
**Reproduction of “The Anatomy Lesson of Dr. Nicolaes Tulp” (1632), Rembrandt van Rijn:'s-Gravenhage, Mauritshuis, Netherlands**. Image in public domain.

The more serious attack on this approach has been conceptual. It is argued that a single rating of esthetic quality does not adequately capture the human understanding of, and response to, works of art. In designing a recent study of our own (Huang et al., 2011), this seemed to be an overriding reason for not pursuing that line of enquiry. For example, some works of art gain their dominance and power because of the strength of the image, rather than its beauty alone (Figure 2). We do not look at the “The Anatomy Lesson of Dr. Nicolaes Tulp” by **Rembrandt van Rijn** just to admire the beauty of the corpse. Rather we are drawn into this scene of flesh and corporality also to look at the status of the members of the surgeon's guild and Dr. Tulp himself, particularly comparing them with the unfortunate who has recently died by execution. As has been pointed out many times, from many different sources of evidence—for example IJpma et al. (2006)—this is not a realistic depiction of an anatomical dissection. Thus, this artwork is not a simple piece of photographic reportage. Neither has it been created just to produce a beautiful object for modern-day viewers to contemplate in an art gallery. At least one purpose in its time was to memorialize the persons in the picture, who can all be identified by name—information, which is carefully embedded for us within the picture, written upon the piece of paper held by the man at the back. So this picture, like many others, is not photographically naturalistic; the scene has been dramatized and arranged to gain impact and significance on behalf of those commissioning the original artwork.

KEY CONCEPT 2. Rembrandt van Rijn Rembrandt van Rijn (1606–1669) is one of the most famous Dutch artists of all time. Working in Amsterdam, he is particularly known for his portraits.

From the point of view of the biologist then, the understanding of the significance of artworks might appear to become more of a question of sociobiology rather than a pure visual analysis of the optical content of the artwork. In the strictest form, this would suggest that the conceptual approach to artwork should be taken as dominant. One would then conclude that any comments made by biologists should be focused on the thought patterns leading up to production of artworks, rather than the visual content of the artworks. This is certainly an important warning against naïvely looking at artworks as visual objects, without considering how and why they came to be created. On the other hand, the artworks we are considering here, specifically the Rembrandt portraits used in our study, are intended to be viewed, as opposed to being heard or read, so the choice of a visual medium for expressing the artist's ideas and feelings is thoroughly embedded in the production.

## The visual content of art

Whilst we have already set aside the goal of a direct assault on measuring correlates of the esthetic content of artwork, arguing that esthetic content is not a single scale of beauty, the question remains of whether art differs visually from “non-art” and, if so, in what way does it differ. It may be of course that such differences do not really exist, at least not in a categorical form.

A number of direct attempts have been made, either to classify art according to broadly defined historical styles (Wallraven et al., 2009) or to test whether an artwork is genuine or fake (Gerhard and Bethge, 2014), a question closely related to the one explored below. These approaches use statistical classification, either to train a machine-learning computer algorithm or to train human observers, to discriminate or categorize digital images of artwork. As an exercise in demonstrating what information may be available from digitized versions of artworks, these approaches are very valuable. For example, such methods may well reveal differences, to which expert viewers are sensitive but find the nature of those differences to be very difficult to articulate.

These approaches are limited however. They rely very largely on low-level details within the images, such as the texturing or color palette in use. Notably, these approaches lack semantic content, so it is difficult to see that they will capture all the dimensions of artwork that would be of interest. Their strength is similar to analytical methods that rely on spectroscopy of pigments, X-ray photo of images that underlie the surface or analysis of canvas or other incidental materials. These measurements are objective, thus usefully discriminating one art object from another, but they do not reveal much about the existence of any critical differences between art and non-art.

Another reference point for considering the visual content of artworks is the stream of everyday images that are formed on our retinas. This visual content has been analyzed extensively, largely because there are strong theoretical grounds for expecting that our visual systems have been adaptively shaped to deal with the statistics of so-called natural images (Ruderman and Bialek, 1994; van der Schaaf and van Hateren, 1996; van Hateren and van der Schaaf, 1998; Koenderink and van Doorn, 2003). Since a good deal of the statistics of the natural world images is shared with the statistics of those artificial images which are artworks, little progress has been made in revealing anything special about art through this route. Indeed, it is possible to invert the explanatory reasoning here and suggest that if the visual system is indeed adapted to the statistical content of natural images, then successful artwork might well have very similar statistical content simply because the artwork is being designed for viewing by visual systems adapted in this particular way.

## Neuroscience as a methodological tool

These various points led us to advocate a position in which, rather than using neuroscience to attempt to supplant earlier and more established forms of scholarship, neuroscience might be put to better use, if it were to address questions that are interesting to art historians and connoisseurs. These would be questions that are regularly debated and figure centrally in discussions about art works. An example of this is the assignment of **authenticity**: in other words, “Is this artwork a genuine Rembrandt, Picasso, Goya?” This is a question that art experts are regularly asked to resolve. A wide variety of techniques and disciplines contribute to these decisions: these range across “hard science” measurements of chemistry of pigments, historical documents about the art work, identification of interior or exterior locations of the artist's activity, comparative judgments with established artworks from the same artist and so on.

KEY CONCEPT 3. Authenticity Authenticity is the degree of confidence provided by art experts that a work of art has been genuinely created and prepared by a known artist. Artworks may be not authentic for a variety of reasons, such as similar artworks by lesser-known contemporaries of the artist, copies of artworks made without any intent to deceive and fakes where a forgery has been attempted.

Decisions about authenticity are not just significant for the experts in ensuring that the history of their field is correctly written. A discovery of a new artwork by an established famous artist is an event that often captivates the headlines of the media and has a profound impact on the monetary value of the artwork and its public esteem. This public esteem is something that drives a great deal of human activity, well beyond a simple auction fight to own a genuine masterwork. As noted earlier, people expend a great deal of time, resources and effort in viewing original authentic artworks in galleries, located far from their homes.

## The gallery experience

Visiting galleries can appear to be a deeply puzzling activity, since the reasons why the actual experience is in any way satisfying can be remote. Consider the gallery at the Louvre where the Mona Lisa is located: the room is typically full with people, such that the view of the artwork is limited; many people view the painting through the medium of their digital camera; the painting is itself protected with bullet-proof glass. Despite all these limitations, there is no lack of new enthusiasts who clearly desire the experience of walking into the gallery in which the authenticated masterpiece is located. In the gallery, the label on the wall next to the painting, which summarizes the combined views of many scholars, assigns huge power and significance to the art object that it sits beside. Whilst viewing the Mona Lisa itself is almost a caricature of the gallery visit, the power of viewing the authentic work is the experience we wished to tap into in designing our study. Authentication of artworks by experts has a clear influence on the experience expected and felt during gallery visits: we wondered what the consequence of such attribution might be within a brain imaging experiment (Huang et al., 2011).

Clearly, bringing the experience of viewing out of the gallery into the laboratory carries with it a number of limitations. One can at least maintain the experience of viewing major artworks by a world-renowned artist, but many aspects of the viewing experience are markedly different (Nessim, 1997). The images are presented on a computer/TV display, so they can never be identical with viewing the real artworks in the controlled lighting of a gallery. The person lies supine in a brain scanner to view the images and the advice about whether the image is a rendition of an authentic artwork is delivered aurally, to avoid any visual stimulation associated with the content of the advice. This means that the observer is more a passive receiver of visual content rather than an active explorer within a large physical environment.

Before becoming too concerned about the effect of removing artworks from galleries on the viewers' experience, it should be recalled that not all artworks were created with the intention of displaying them in an art gallery, as conceived in the modern sense. The Anatomical Lesson (Figure 2) was commissioned so that it could be exhibited within the Guild Room of the Amsterdam Guild of Surgeons. It is also surprising but true that the time spent by individuals in looking at individual paintings in a gallery setting is quite short, some 15–30 s being typical (Smith and Smith, 2001). Hence, presentation of a controlled sequence of images for durations similar to these will not be far removed from the temporal sequences chosen by many viewers in the gallery context. Another approach, which allows working in the gallery space itself and we have implemented in other work (Khoshdel, 2012), is to make use of recent developments in portable monitoring equipment, which can take psychophysiological measurements (heart rate, breathing, skin conductance response, eye and head position to measure gaze) as a person is moving freely (Locher, 2011).

## Experts and their training

One of the more fundamental decisions to be made in any study of this kind is to consider who will be the participants. There are strong arguments for exploring responses of expert viewers, as in Pihko et al. (2011). Experts may be expected to be the most highly trained viewers of the artwork and therefore they might exhibit the most highly differentiated brain responses. The main methodological issue about studying the brains of experts is that their expertise is often highly particular. This is especially true in the art field, in which some experts may be driven by their knowledge of the history of the times and the cultural significance of the artwork in that context, whereas other experts may have detailed technical knowledge about the methods of preparation and construction of the work of art. At least some expertise is difficult, sometimes impossible, to articulate: an example very often quoted is that of determining the sex of day-old chickens, which has for many years been a highly specialized employment (Biederman and Shiffrar, 1987). This means that it can be difficult to gather together a group of individual experts who hold a consistent domain of expertise.

Even the terminology is apt to be confusing here. One recent research study Pang et al. (2013) defined expertise with reference to a questionnaire that certainly evaluates awareness of and sensitivity to the production and evaluation of artwork, but does not probe differences as subtle as those between a specialist in Rembrandt and a specialist in the Impressionists. Indeed, the general outcome of that questionnaire is that a fraction of the people tested in Huang et al. (2011) would be “expert” by the criteria of Pang et al. (2013). The alternative approach attempts to embrace the particularity of expertise; for example, by arranging for brain scanning of an individual artist during the production of artwork and comparing the brain activations with those from a “non-artist” (Solso, 2000). Although this work is pioneering, studies of this kind will have to embrace understanding about the nature of expertise from other research in psychology and neuroscience, as well as a considerable improvement in the reliability of signals from human brain scanners, before progress can be made.

For our own study, fortunate timing was offered by the current state-of-affairs in the scholarship of Rembrandt portrait paintings. There has been a fundamental restatement of attributions of artworks to Rembrandt in the last few years (van Sonnenburg, 1995; Seinstra, 2010). To all but the experts, the decision about which is a “real Rembrandt” and which is a fake is a matter on which recent advice rather than past knowledge must be predominant. There are several portrait paintings that have had until recently the status of genuine works by the great master but are now regarded as inauthentic at some level or other. These circumstances make it relatively easy to create an experimental design in which some participants experienced a painting under the advice that it is genuine whilst others receive the advice that the same portrait is a fake. (No person saw the same portrait painting twice under different advice conditions.) Since the context is important, we carefully prepared a script for each participant that explained what was meant by “fake,” noting for our study that this everything that is not genuine: misattributed works; copies made by admirers of Rembrandt with no intent to pass them off as genuine; as well as outright forgeries. The same script delivered historical information about Rembrandt and his artworks, with the intent of bringing participants' knowledge to a level playing-field.

The circumstances created by the significant number of Rembrandt portraits and the uncertainty surrounding their authenticity created a special opportunity. It was for this reason and a number of others (related to the complexity of the pictorial content) that we avoided the use of artworks such as that the Rembrandt in Figure 2. It seemed to us that the overriding issues is that it is difficult to design experiments in which all members of a group of participants have similar levels of expertise. By its very nature, expertise varies with the past history of the individual. Nonetheless, understanding the nature of expertise, particularly as it applies directly to the appraisal of artwork, is a question that is of interest to the art professionals and historians.

## Brain regions involved in responding to authenticity

Neuroscientists have identified a number of substantive divisions of function between different areas of the human brain. Until fairly recently, this work progressed by the patient acquisition of insights from studying individuals who have suffered damage to specific regions of the brain. Nowadays, the use of modern imaging techniques has greatly accelerated this field of activity. One of the questions that was open before our study is whether there are separate, as yet unidentified, regions of the brain that would be responsive in our task involving artwork.

Broadly speaking, this question was answered in the negative. Whilst there are interesting activations in response to the instruction that “this painting is authentic” and separate activations in response to the opposite instruction that “this painting is a copy,” the brain regions involved are fairly familiar. When the “authentic” instruction was issued, the **orbitofrontal cortex** was mildly activated. This cortical region has been identified as involved in the receipt and acknowledgment of rewarding stimuli, in a variety of contexts from pleasant-tasting food (Rolls, 2000) to gambling tasks (Camille et al., 2004). This is consistent with the idea that the subjects in our task found it more rewarding to view an image proclaimed as a genuine, authentic Rembrandt, as opposed to an image that was labeled as derivative.

KEY CONCEPT 4. Orbito-frontal cortex Orbito-frontal cortex is part of the cerebral cortex close to the front of the head known to respond to highly rewarding stimuli.

The activations generated by the advice that the image is a “copy” were more complex. A number of brain regions (pre-cuneus, middle frontal gyrus, and **fronto-polar cortex**) were involved, but the most striking of these was the activation of fronto-polar cortex, as its activation is given some insight by considering the verbal reports of our participants. They mentioned that the advice that the artwork was a “copy” reliably induced within them a train of thought that questioned in which regard the displayed artwork was less than genuine. In other words, they began to run through a set of possible options or hypotheses that would reveal the works as fake. This is the sequence of thought processes that has been shown in other contexts to unleash fronto-polar activations (Koechlin and Hyafil, 2007), as policy options are evaluated and discarded.

KEY CONCEPT 5. Fronto-polar cortex Fronto-polar cortex is a part of the cerebral cortex that lies at the front of the head in the part of the skull just above the eye sockets. The functions of front-polar cortex are still poorly understood, but it is regarded as important in the ability to engage in high-level cognitive planning and strategic thinking.

Most interesting were the activations of the **occipital cortex**. Here there was no significant difference in activation between the two conditions in which different advice about the artwork was issued. Since the occipital regions are very much the primary input to the brain for visual information, this suggests that there was no fundamental difference in the effect of the advice on the visual responses. As well as standing in its own right as an observation, this also suggests that neither of the two different conditions unleashed different levels of attention, which if present would expect to be manifest at the level of occipital cortex. What did happen however was a closer coupling between the activations of fronto-polar cortex and occipital cortex during the “copy” condition: the response of each region is variable but under the “copy” condition there is a greater tendency for the two regions to vary together (co-vary). We cannot determine a direction of causation from this type of measurement, but it is reasonable in the light of other knowledge about the functions of these brain regions to view this enhanced co-variation as being driven from fronto-polar cortex to occipital cortex. On this view, the enhanced co-variation would be a correlate of the hypothesis-sifting activity of fronto-polar cortex, as it sends signals to occipital cortex to validate or reject the currently active hypothesis.

KEY CONCEPT 6. Occipital cortex Occipital cortex is a part of the cerebral cortex that lies at the back of the head underneath the occipital bone. This region of cortex contains the major cortical sites involved in processing visual information.

Our results therefore highlight two points. First, the brain regions activated in some aspects of the appreciation of art, specifically its authenticity, are also involved in many other cognitive and emotional responses. This emphasizes the continuity between the appreciation of art and other human cognitive behavior, rather than regarding the appreciation of visual art as being a human activity that is unique and may be therefore expected to have distinct and separate brain regions associated with it. Second, the responses in the “copy” condition, which involve close co-ordination between brain regions that are widely separated anatomically, emphasize the significance of whole brain, integrated responses to artworks. Specifically, in this case, the interaction is not manifest in the response of a single brain region, but requires analysis of the co-variation of activity between different brain regions to reveal this. Whilst our experiments do not directly address the question of whether there is a brain region, whose activity correlates with the esthetic appreciation of artworks, it is evident from observation of human activity alone that the search for viewings of authentic works is a major influence on the esthetic response. The fact that the brain responses here are manifest by means of an interaction between brain regions, rather than the activation of a single brain region, suggests that other esthetic responses may depend upon coherent activity across many areas of the human brain.

## Dividing the authentic from the fake

As a footnote, the following point may be noted. Within our study is also the capacity to ask a significant question about the authenticity of the artwork itself. Recall that the design of the experiment resulted in each image, which was either real or fake, being viewed under two conditions, with advice that it was authentic or the opposite. Although the people taking part in the study showed no evidence of being able to distinguish the real from the fake artwork, we wondered whether their cortical responses might be able to signal something that they could not report explicitly. Discovering of a reliable way of distinguishing real from fake artwork would of course make us rich beyond the dreams of academics! However, we found only the slightest difference in cortical response that distinguished the artwork itself. This difference was confined to the occipital regions, as opposed to the activations induced by advice about authenticity of the artworks, which was described earlier. Curiously, this small differential response was primarily due to a difference in the responses of left and right occipital cortex, strongly suggesting that this differential response was driven by low-level differences in the image structure of the artwork. In the case of portraits, this would be for example a difference in the left-right symmetry of the images that were used in our study. This is an important methodological issue for the use of artwork in brain imaging studies, which is that it is very difficult to match a meaningful stimulus set at all possible levels of description. The use of portraits in one artistic style reduced these incidental differences as much as possible.

## Conclusion

It is perhaps unsurprising that what people themselves think about art has an influence upon their brain responses. What has been shown here is that what people are invited to think about art also has profound effects upon their brain responses. The conduct of imaging studies inevitably produces many possible factors that need to be appreciated and brought under experimental control. In designing future neuroscience studies about the human response to visual art, it becomes important to plan for and embrace the reality that even non-directed and innocently provided forms of advice may have a profound effect on the brain responses of participants. Whilst the research undertaken here has probably revealed little about the response of people to the art of Rembrandt, the use of portraits, being Rembrandt's most intimate and personal works, has clearly had a significant impact upon our viewers, even though they have been spatially confined to the interior of an MRI scanner at the time of viewing. The impact of the advice about authenticity would presumably have been far weaker, if the same advice had been issued about unknown artwork of little cultural significance.

### Conflict of interest statement

The author declares that the research was conducted in the absence of any commercial or financial relationships that could be construed as a potential conflict of interest.
